# Selective and deceptive citation in the construction of dueling consensuses

**DOI:** 10.1126/sciadv.adh1933

**Published:** 2023-09-22

**Authors:** Andrew Beers, Sarah Nguyễn, Kate Starbird, Jevin D. West, Emma S. Spiro

**Affiliations:** ^1^ Department of Human Centered Design and Engineering, University of Washington, WA 98195, USA.; ^2^ Information School, University of Washington Seattle, WA 98195, USA.

## Abstract

The COVID-19 pandemic provides a unique opportunity to study science communication and, in particular, the transmission of consensus. In this study, we show how “science communicators,” writ large to include both mainstream science journalists and practiced conspiracy theorists, transform scientific evidence into two dueling consensuses using the effectiveness of masks as a case study. We do this by compiling one of the largest, hand-coded citation datasets of cross-medium science communication, derived from 5 million Twitter posts of people discussing masks. We find that science communicators selectively uplift certain published works while denigrating others to create bodies of evidence that support and oppose masks, respectively. Anti-mask communicators in particular often use selective and deceptive quotation of scientific work and criticize opposing science more than pro-mask communicators. Our findings have implications for scientists, science communicators, and scientific publishers, whose systems of sharing (and correcting) knowledge are highly vulnerable to what we term adversarial science communication.

## Teaser

A large dataset of Twitter arguments about masks is used to show how consensus is formed in the public eye.

## INTRODUCTION

Public consensus often diverges from scientific consensus (*1*, *2*). Something is lost in translation from the scientist to the public, as “science communicators,” writ large to include both mainstream science journalists and practiced conspiracy theorists, reshape and transform scientific evidence in ways that may deviate from its authors’ intents (*3*). For example, in 2015, a paper suggested that cloth masks increase the risk of respiratory illness in hospital contexts (*4*). It became one of the most shared papers about masks on social media during the coronavirus disease 2019 (COVID-19) pandemic, was quoted in one of the most popular television news programs in the United States, and was cited in legal challenges to mask mandates (*5*, *6*). However, the paper’s first author wrote more than 10 papers over a 2-year period during COVID-19 affirming the benefits of masks and speaking out against the selective and misleading understanding of the 2015 paper, before and after its popularity on anti-mask media (*7*–*16*).

How does this reimagining of scientific consensus outside of the hands of scientists themselves happen in today’s media-rich environment? What are the implications, and what can be done about it?

Here, we hand-curate one of the largest corpora for investigating these questions. Leveraging the high demand for research findings about masks during the early stages of the COVID-19 pandemic, we show via citation analysis how science communicators and practiced conspiracy theorists alike manufacture the appearance of consensus using selective and critical citation to create dueling, opposite perceptions of scientific knowledge. We find that scientific publishers and authors are unprepared for how their work is being misused by science communicators to advance scientific misunderstanding.

### Science communication during a pandemic

Rarely have so many people been so personally invested in emerging scientific discoveries, and never before has the process of science communication been so thoroughly documented on social media as it has during the COVID-19 pandemic. These circumstances have shown vividly how varied the sources of scientific knowledge have become: not only scientists but also journalists (*17*), government officials (*18*), celebrities (*19*), viral videos (*20*), and social media users themselves (*21*, *22*). We present and analyze here two of the largest hand-coded datasets of scientific citation to understand the development of a pandemic controversy around masks in the United States. The first dataset shows how scientists built and shared among themselves a varied evidence base on the effectiveness of masks for COVID-19. The second dataset shows how science communicators, credentialed and not, transformed this evidence base into two different, opposing perceived consensuses: one in favor of masks and one against. In documenting this process of consensus transformation, we have three primary findings:

1) While scientists largely agreed that masks were effective at reducing the spread of infectious disease and tended to cite works with similar methodologies, science communicators sharply disagreed and tended to cite work with similar stances.

2) While scientists rarely engage in negative citation, science communicators and particularly anti-mask science communicators more frequently disparage and criticize scientific work.

3) Science communicators cite much of the same literature as scientists. However, consensuses diverge via misleading contextualizations that often contradict the scientists’ publicly stated views.

While popular rhetoric has held that those who disbelieve mainstream science are “anti-science” (*23*, *24*), our dataset describes a communicative world where both pro- and anti-mask social media users are attuned to scientific norms and invest heavily in citing the primary literature (*25*). In what is akin to context collapse (*26*), anti-mask science communicators gladly repurpose the knowledge infrastructure of mainstream science to spread misinformation in the language of science itself (*27*). These science communicators’ frequent use of negative citation (*28*) can be seen as inoculating their audiences against the views of mainstream science, the mirror of a strategy of “prebunking” and “inoculation” advocated by science communication researchers (*29*–*31*). Anti-mask science communicators’ deceptive but likely effective citational practices reveal how scientific authors and publishers are currently woefully unprepared for the ways that their articles are misrepresented to the public (*32*).

Conceptually, this work bridges consensus research in science communication, which focuses on the public communication of science (*1*, *33*–*35*), science and technology studies, which focuses on how consensus is constructed and then understood by both scientists and the public (*36*–*38*), and the science of science, which focuses on consensus formation through citational networks (*39*–*42*). We focus on the role of formal (government health bulletins) and informal (anonymous viral Twitter posts) science communication. The work is inspired by the concept of knowledge infrastructures: the “robust internetworks of people, artifacts, and institutions which generate, share, and maintain specific knowledge about the human and natural worlds” (*43*). By centering both formal and informal knowledge creators as objects of analysis, we create opportunities for quantitative and qualitative inspections of knowledge infrastructures and the way they shape public health outcomes.

## RESULTS

### The creation of evidence bases by scientists

In these findings, we make a distinction between scientists, who publish their work for consideration by the scientific community, and science communicators, who publish their work for consideration by the lay public and may or may not include formally trained scientists. We assume that the goal of science communicators in crises is to translate the (sometimes developing) consensus of scientists into public-facing information about probable risks and recommended responses (*44*). In practice, we find that popular science communicators in the United States are sharply divided on the benefits of masks for preventing COVID-19. Thus, our first task is to understand whether these science communicators are faithfully transmitting a true dissensus among scientists themselves or are generating it from whole cloth.

**Fig. 1.**
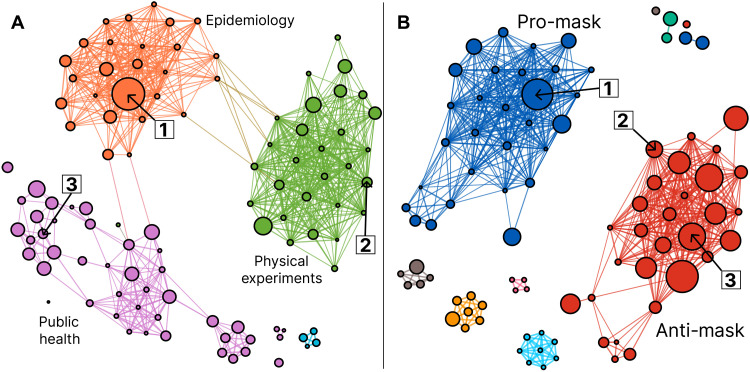
A visualization of the cited literature of scientists and science communicators with respect to mask wearing and COVID-19. Papers that tend to be cited together by (**A**) scientists and (**B**) science communicators discussing masks and COVID-19. Scientists tend to cite groups of papers by topic or field. In (A), the green cluster represents papers concerned with the physical properties of mask materials; the orange cluster represents epidemiological papers on masks effectiveness against COVID-19, and the purple cluster represents older public health papers on the effectiveness of masks in hospitals and households for COVID-19 and other respiratory illness. In (B), meanwhile, science communicators cite largely the same papers as scientists but segregate their citations by stance: supporting the use of masks in blue and opposing them in red. Three papers (nodes) are labeled in both clusters: (1) (*62*), the most shared paper in both datasets, (2) (*89*), a fluid physics paper from 2010 shared in the anti-mask perceived consensus, and (3) (*4*), a 2015 paper with a negative finding on masks whose lead author has since publicly supported mask wearing for COVID-19. Larger nodes in the scientist network (A) are cited by more highly cited papers on masks, while larger nodes in the science communication network (B) are cited by more frequently shared Twitter URLs in a dataset of people arguing about masks.

To assess the existing evidence base on mask wearing at the start of the pandemic, we use the Web of Science (*45*) to identify papers about masks that were highly cited in the first year of the COVID-19 pandemic. We annotate all citations in these papers and mark whether they are positive/neutral citations or negative citations. We define negative citations as citations that go beyond discussing a work’s limitations or conflicting results to actively recommend that readers at least partly disregard the cited work. We construct a signed (i.e., positive and negative citations specified through hand coding) cocitation graph of these highly cited papers to identify subsets of papers that tend to be cited together (Fig. 1). We proceed under the assumption that papers frequently cocited positively together, within a topical area, represent endorsements of shared evidence on the topic of masks in COVID-19 (*46*).

We find three evidence bases that correspond to different methodologies and fields that highly cited masks papers tend to reference. Specifically, these papers cite (i) studies grounded in particle physics that evaluate the material properties of masks, (ii) studies grounded in epidemiology, which evaluate masks effectiveness on different metrics during the first year of COVID-19, and (iii) studies grounded in public health, which evaluate the effectiveness of masks in different contexts, such as hospitals or households, and evaluate practical considerations such as the disinfection of masks. Each evidence base is composed of papers that mostly suggest that masks are effective for preventing respiratory illness and especially COVID-19.

We find that, in general, scientists use negative citation sparingly, with only 1.3% of citations across 80 papers manually coded as negative (32 of 2426 citations). This percentage is notably lower than previous estimates, which have ranged from 2 to 15% (*28*, *47*, *48*). Affirming Bruggeman *et al.* (*39*), we find that negative ties, while rare, are not randomly distributed throughout the citation corpus. Rather, they are disproportionately directed at a few papers, including both papers with positive and negative results toward masks. The most negatively cited paper, with four negative citations, is MacIntyre *et al.*’s 2015 paper in *BMJ Open* that “cautions against the use of cloth masks” for preventing respiratory illness in hospitals and suggests that they may increase the risk of infection to the wearer (*4*). This paper is particularly notable because its lead author has subsequently published no fewer than 10 publications over a 2-year period arguing for the use of masks of all types to lessen the spread of COVID-19 (*7*–*16*). Among these publications is a letter titled “Masks in the community are an effective strategy” and a follow-up to the criticized 2015 study offering a possible explanation for their earlier, negative findings around cloth masks (*11*, *13*). Chu *et al.*’s 2020 paper claiming that “face mask use could result in a large reduction in risk of [respiratory] infection” was also negatively cited three times primarily on methodological grounds, some of which are described by Jüni *et al.* in a letter response published in the same journal (*49*, *50*). Overall, scientists negatively cite papers that have perceived methodological weaknesses rather than papers that share a certain stance on the effectiveness of masks.

In this first year of the pandemic, the consensus on the effectiveness of masks was still developing, and new studies continue to be published since our analysis period. We show here not a proof of consensus that mask wearing is an optimal choice for combating COVID-19 but rather the creation of a shared literature base from which scientists, within their own fields, collectively endorsed and used to move scientific knowledge and work forward. In the following section, we describe the different way that science communicators select literature bases, namely, by their ability to support a pro- or anti-mask stance.

### The creation of perceived consensuses by science communicators

**Fig. 2.**
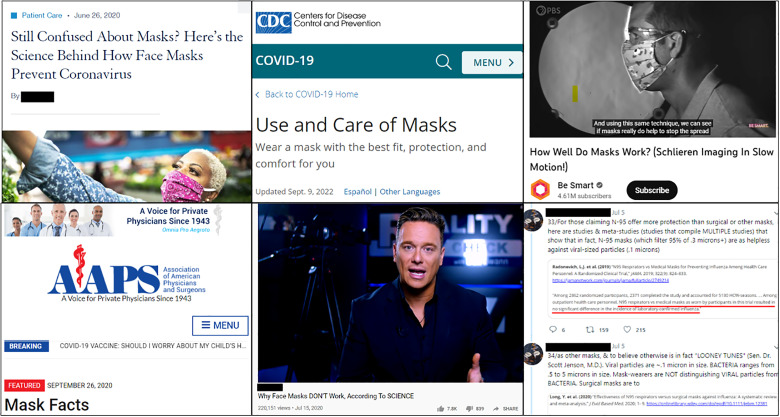
Screenshots of six popular science communicators in our dataset. The top row are examples of generally pro-mask science communicators, including, from left to right, an article published by the University of California San Francisco (UCSF), a help page published by the CDC, and a YouTube video published by the PBS. The bottom row contains generally anti-mask science communicators, including, from left to right, a page published by the medical conspiracy group, the Association of American Physicians and Surgeons (AAPS), a YouTube video published by a conspiracy theorist formerly employed by Russian state media RT, and a viral threaded Twitter post by an anonymous user containing URLs and screenshots of scientific articles. We have appended black boxes to the first image in the top row, as well as the second and third images in the bottom row, to remove names of individual authors.

Science communicators, by contrast, come to remarkably diverging conclusions about the effectiveness of masks within their own community. To surface the published works of science communicators, we analyze the URLs shared in posts by Twitter users discussing masks. These URLs sometimes link directly to the published scientific literature, but more often, these URLs link to the works of science communicators, broadly defined: science journalism articles, government- and hospital-sponsored websites, self-published personal blogs, viral social media posts, and videos hosted on a variety of platforms. Some examples of frequently shared URLs that support mask wearing include, with screenshots in the top row of Fig. 2, the following: (i) “Still Confused About Masks? Here’s the Science Behind How Face Masks Prevent Coronavirus,” hosted by the University of California San Francisco (UCSF) Medical School (*51*), (ii) “Guidance for Wearing Masks” hosted by the U.S. Centers for Disease Control and Prevention (CDC) (*52*), (iii) a video hosted on YouTube titled “How Well Do Masks Work? (Schlieren Imaging In Slow Motion!)” published by the U.S. Public Broadcasting Service (PBS) (*53*), and (iv) a long, threaded Twitter post that aggregates links to scientific studies purportedly supporting masks, created by a pseudonymous Twitter user (*54*).

Conversely, examples of highly shared URLs that oppose mask wearing, again with screenshots in the bottom row of Fig. 2, include the following:

1) “Mask Facts,” a website hosted by the Association of American Physicians and Surgeons (AAPS), a medical conspiracy group aligned with the U.S. libertarian movement, which opposes the scientific consensus on vaccines, HIV/AIDS, climate change, and abortion among other topics (*55*).

2) “Masks Don’t Work: A Review of Science Relevant to COVID-19 Social Policy,” a self-published literature review authored by a scientist who also contested the scientific consensus on climate change and vaccines. This review has been rehosted on many different websites, including the science aggregator ResearchGate (*56*).

3) A video hosted on YouTube titled “Why Face Masks DON’T Work, According To SCIENCE” created by a journalist then employed by RT, a Russian state-owned news outlet known to publish false and misleading content. The author advocates for counterconsensus views on vaccines and a variety of conspiracy theories related to mass shootings in the United States, the Syrian Civil War, and other topics (*57*).

4) “The Science of Masks,” a website and companion video that links to more than 60 studies and news articles about masks. It is hosted on the website of an alternative medicine podcast host that sells diet planning services, books about wellness, and speaking opportunities (*58*).

5) A long, threaded Twitter post that aggregates links to scientific studies and other materials purportedly opposing masks, created by a pseudonymous Twitter user (*59*).

The science communicators behind each of these websites, whether pro- or anti-mask, explicitly cite peer-reviewed scientific literature to explain their positions on the effectiveness of masks. Anti-mask science communicators cite the scientific literature in ways similar to pro-mask communicators, for example, by using explicit “References” sections with formatted citations. They cite nonscientific sources, such as personal blogs or anecdotal news stories, only rarely. Most anti-mask communicators use sophisticated, scientific language to contextualize their findings, for example, referring to “randomized controlled trials” and “statistical significance,” and likely give the impression of a knowledgeable expert explaining the current state of scientific consensus.

To understand how science communicators reference science differently from scientists, we code their citations just as we did with the Web of Science data. We find that the proportion of negative citations is notably higher from science communicators than scientists themselves at 6% (89 of 1496). This proportion rises to 9% for websites opposing or reporting negative findings on masks compared to 4% for websites with neutral or positive findings. Overall, anti-mask websites averaged 1.5 negative citations per website, while other websites average 0.7 citations. Several anti-mask websites have sections specifically devoted to refuting research that supports masks. For example, one such section is titled “Popular Mask Effectiveness Studies That Ignore Real World Conditions,” and lists the titles of pro-mask papers accompanied with short explanations of their supposed flaws in scientific language.

As with the Web of Science data, we use this dataset to create a signed cocitation network (Fig. 1). We see several small groups of two to seven papers, each likely the result of different science communicators using extremely similar citation lists or, in some cases, directly copying and excerpting each others’ pieces. The rest of the graph is dominated by two large groups of papers that, unlike citations in the scientific literature network, group together by stance. One group is pro-mask and consists entirely of literature from mostly high-impact scientific journals whose results support the claim that masks are at least somewhat effective for preventing the spread of COVID-19 or other respiratory illnesses. A typical example of a paper in the pro-mask evidence base is that of Leffler *et al.* (*60*), which states in its abstract that “societal norms and government policies supporting the wearing of masks by the public, as well as international travel controls, are independently associated with lower per-capita mortality from COVID-19.”

The other group is anti-mask and consists of the scientific literature and one university-affiliated website with positive, qualified, or negative findings about masks, almost all of which are construed to appear as negative findings in the context of their citations. A typical example in this cluster is the work of Jacobs *et al.* (*61*), which argues in its abstract that “face mask use in health care workers has not been demonstrated to provide benefit in terms of cold symptoms or getting colds.” Both groups of papers contain almost exclusively papers published in academic journals, and neither cluster displays a clear preference for papers from the fields or journals identified in the scientific literature network.

Both the pro- and anti-mask paper groups leveraged papers from the three different fields found in the scientific literature, extracting those that can be made to appear in agreement with their chosen stance. Some papers are cited positively by both pro- and anti-mask science communicators: Fifteen percent of incoming citations to the pro-mask cluster come from websites with anti-mask orientations, and 23% of incoming citations to the anti-mask cluster come from websites with pro-mask orientations. However, rather than take these cross-stance citations as evidence of shared scientific knowledge between pro- and anti-mask perspectives, we find that papers are often contextualized differently between pro- and anti-mask websites (see Table 1 for excerpts). Accordingly, the separation between the evidence bases that readers perceive may be even wider than these data can illustrate. We discuss two such papers at length to illustrate two distinct tactics by which ostensibly pro-mask papers can be contextualized to appear to advise against masks: selective quotation and deceptive quotation.

**Table 1.** A list of the top five most-shared citations (measured by number of shares of sources that cited them) in the Twitter dataset. We provide examples of positive and negative framings for each of the papers’ results/conclusions.

Paper citation	Pro-mask context	Anti-mask context
Leung *et al.* (*62*)	“It found that even loose-fitting surgical masks blocked almost all the contagious droplets the wearers breathed out and even also some infectious aerosols – tiny particles that can linger in the air.” (*90*)	“Surgical masks can efficaciously reduce the emission of influenza virus particles into the environment in respiratory droplets, but not in aerosols.” (*55*)
MacIntyre *et al.* (*4*)	“One of the most frequently mentioned papers evaluating the benefits and harms of cloth masks have been by MacIntyre et al. Findings have been misinterpreted, and therefore justify detailed discussion here... Its implementation does not inform the effect of using cloth masks versus not using masks in a community setting for source control of SARS-CoV-2, which is of the same genus as seasonal coronavirus, which has been found to be effectively filtered by cloth masks in a source control setting.” (*79*)	[Transcribed from video] “Now here’s what’s even more shocking about the study. Not only were the rates of infection higher in the cloth mask group than they were in the medical mask group. The rates of infection in the cloth mask group were significantly higher than the people in the control group, who are allowed to occasionally wear a mask or not even wear a mask at all.” (*58*)
Long *et al.* (*64*)	“Further, the evidence is mixed from randomized studies on types of masks and risk for influenza-like illness transmission to mask wearers; for example, a recent systematic review and meta-analysis comparing N-95 respirators versus surgical masks found a statistically insignificant decline in influenza risk with N-95 respirators.” (*91*)	“There were no statistically significant differences in preventing laboratory-confirmed influenza, laboratory-confirmed respiratory viral infections, laboratory-confirmed respiratory infection, and influenza-like illness using N95 respirators and surgical masks.” (*56*)
Radonovich *et al.* (*92*)	“Radonovich et al. found in an outpatient setting that use of N95 respirators, compared with medical masks... resulted in no significant difference in the rates of laboratory-confirmed influenza.” (*79*)	[Transcribed from video] “In the 2019 study, there was no significant difference by wearing an N95 respirator mask.” (*57*)
Smith *et al.* (*93*)	“If someone asks: What’s the evidence for mask wearing? Here is a list of *SEVENTY* papers, including reviews/meta-analysis and individual studies, in reverse chronological order. Includes 31 from 2020 alone (!!). META = meta-analysis or systematic review.” (*54*)	“This 2016 meta-analysis found that both randomized controlled trials and observational studies of N95 respirators and surgical masks used by healthcare workers did not show benefit against transmission of acute respiratory infections.” (*94*)

We first examine the work of Leung *et al.* (*62*), the paper that was most often cited by science communicators in our dataset. Inspecting how this paper was cited in text, we find that pro- and anti-mask websites selectively quote entirely different parts of the paper. Pro-mask science communicators tend to quote or paraphrase the top-level finding from the abstract: “surgical face masks could prevent transmission of human coronaviruses and influenza viruses from symptomatic individuals.” Anti-mask science communicators instead quote a sentence in the Discussion section, “our findings indicate that surgical masks can efficaciously reduce the emission of influenza virus particles into the environment in respiratory droplets, *but not in aerosols*” [emphasis added, for additional discussion of aerosols and COVID-19, see (*63*)]. In practice, the work of Leung *et al.* exists as two papers with two, seemingly contradictory conclusions: one that claims that surgical masks can prevent transmission of COVID-19 and another that claims that they cannot, given that COVID-19 is an aerosolized disease. We observe that science communicators tend to selectively quote or cite the paper to support their stance on masks.

Next, we look at the work of Long *et al.* (*64*), a paper that is cited positively by both popular pro- and anti-mask websites. Long *et al.* concluded from their literature review on mask wearing that “the use of N95 respirators compared with surgical masks is not associated with a lower risk of laboratory-confirmed influenza” and recommended that surgical masks may thus be a suitable substitute for N95 respirators. In three separate anti-mask websites, their results were quoted out of context as follows: “There were no statistically significant differences in preventing laboratory-confirmed influenza, laboratory-confirmed respiratory viral infections, laboratory-confirmed respiratory infection and influenza-like illness using N95 respirators and surgical masks.” Taken alone, this quotation can make it appear that neither surgical masks nor N95 respirators had any effectiveness for preventing disease, a finding that directly contradicts the main result of Long *et al.*, which is about the effectiveness of N95 masks compared to surgical masks. In this case, quotation is not so much selective as deceptive, implying that surgical masks are ineffective through the removal of additional context rather than that surgical masks are as effective as N95 masks and both of which are associated with lower risk of illness.

We conclude our findings by noting that the anti-mask paper group is not a marginal phenomenon in the realm of Twitter debates, and the websites that cite them are shared more often than those that share the pro-mask paper group. To quantify the difference in how often Twitter users are exposed to these two paper groups via shared URLs, we sum the shares for each cited publication across all the science communicators citing them positively. According to this metric, nodes in the anti-mask cluster are shared positively more often than nodes in the pro-mask cluster (124,000 shares versus 76,000 shares). By this metric, some nodes in the pro-mask cluster have more negative exposure than positive exposure, such as that of Leffler *et al.* (*60*), which is not true for any node in the anti-mask cluster. We echo other researchers’ caution of “digital bias” (*36*), namely, that arguments offered in online forums are not likely to reflect public opinion writ large. Our chosen forum in this analysis, Twitter users arguing with each other in the wake of public health announcements by U.S. governors, likely attracts a unique population subset of politically engaged, internet-savvy commenters. Anti-mask viewpoints may have been specifically elicited by the choice of a highly politicized discussion forum. However, to the extent that U.S. governors’ official announcements on Twitter are meant to be a public and visible forum for discussing the pandemic, responses in the aggregate support the appearance of a misleading anti-mask consensus and oppose the mainstream pro-mask consensus.

## DISCUSSION

We describe how science communicators cite the collected works of scientists differently to create the appearance of two radically different scientific consensuses: one supporting mask use during COVID-19 and one opposing. We find that science communicators perform a transformation of the original evidence bases used by scientists, which are mostly organized by fields and methodologies, into separate evidence bases segregated by stance toward masks. Science communicators, who, on one side, are from mostly mainstream scientific and journalistic institutions and, on the other, are mostly a coalition of conspiracy theorists and creators on alternative media platforms, collect different scientific papers and through a process of aggregation (collection of positive citation) and denigration (negative citation) construct a perceived scientific consensus for their audiences. Some papers are referenced by both pro- and anti-mask science communicators, but rather than indicating shared scientific understanding, we see that these papers are either selectively or deceptively quoted to imply the opposite of their conclusions by anti-mask communicators. Overall, we portray the development of an anti-mask community of belief, which, far from being anti-science as their detractors may claim (*23*), puts concerted (and deceptive) effort into the boundary-work of separating favored “good science” from disfavored “bad science” (*65*). We also contribute a method for mapping the transformation, which occurs from the work published by scientists, so often the stopping point of scientometric analysis, into the consensus that the public perceives in online settings.

We find that negative citation is an important practice in the development of these two separate perceived consensuses on Twitter and that anti-mask science communicators in our data use negative citation more frequently than other communicators. Science communication scholars have argued that prebunking, i.e., telling audiences that they will encounter misinformation, may be an effective way to communicate science and inoculate them against false beliefs (*29*–*31*). We find that, unfortunately, charismatic disinformers also inoculate their readers with critical citations of otherwise widely trusted research. We reflect that mainstream science communicators’ apparent reluctance to cite critically may stem from not wanting to attract attention, even critically, to counterconsensus findings. Although some research supports the benefits of prebunking (*66*), other findings have claimed that the “oxygen of amplification” may ultimately be a negative factor and that counterconsensus sources are best left uncovered by mainstream media (*67*, *68*). Those outside the scientific mainstream, however, need not worry about amplifying their opponents, as they may assume that most people will inevitably encounter the mainstream position. We recommend further research on the circumstances in which it may or may not be advantageous to negatively cite information that audience members may not have previously encountered.

Although we study Twitter here, it is likely that the pattern that we find persists across other forms of media. A quotation from one of the most-shared papers in the anti-mask consensus that we identified, by MacIntyre *et al.* (*4*), was cited in 2021 on what was at the time the most popular broadcast television program in the United States, Tucker Carlson Tonight, and in a 2022 legal brief successfully advocating for the abolition of federal mask mandates on public transportation (*5*, *6*). This is particularly unfortunate because Dr. MacIntyre herself has worked to support mask wearing in many subsequent letters and peer-reviewed publications and even specifically qualified the results of the 2015 paper in question (*13*). While experts in public health have the ability to assess MacIntyre’s 2015 paper holistically in light of her more recent work, non-experts, who encounter the 2015 paper in isolation on the journal’s website or aggregators such as PubMed, likely do not. This phenomenon has previously been called context collapse, where one group of users (non-experts) encounters information meant for a different group (experts) and consequently lacks the resources to correctly reason about it (*26*). A key qualitative result from this work is how unprepared scientific publishers are for the implications of context collapse on their published archives.

To overcome context collapse, publishers could allow experts to append contextual information to papers that have been or are likely to be misused and do so in formats that are difficult for non-experts to ignore, i.e., appending a large, conspicuous warning box rather than the small ones that we find for these papers [e.g., in (*69*)]. Sites that rehost scientific content, such as PubMed and ResearchGate, must show a willingness to propagate such contextualizations, as the content in our study was often linked to these sites. Scientists themselves must show a willingness to create such contextualizations, and scientific institutions must create credit mechanisms that reward the creation of these contextualizations. Scientists’ peers must understand and acknowledge that rather than a show of weakness on the part of the original authors’ work, these additional comments represent valuable public service. Scientists, reviewers, and editors should also take care to craft their papers’ abstracts with specific statements that are not so easily misquoted, as paper abstracts are sometimes the only information available for lay readers without subscriptions to closed-access research. Such interventions can make the knowledge infrastructure of science more resilient to misuse by bad actors while preserving the value of that infrastructure to expert scientists. We encourage additional research into what forms such interventions may take and which are effective in defanging the work of counterconsensus science communicators (*70*).

The implication of this work that consensuses are socially constructed by non-expert mediators may cause familiar anxiety among scientists, as it can suggest that mainstream scientific consensus is non-objective and thus potentially just as untrustworthy (*71*) as alternative consensuses. We take the perspective of Oreskes (*37*) in her work *Why Trust Science* and believe that the selection of a certain consensus should depend on the diversity and strength of the vetting system that a consensus’s authors practice. The architects of the anti-mask consensus found here are largely an informal coalition of conspiracy theorists, creators on alternative media platforms, and others who have previously shared false theories about climate change, the benefits of vaccination, the existence of mass shootings, HIV/AIDS, abortion, the reality of the moon landing, and a variety of other topics. Few have any scientific background in mask effectiveness or even public health more generally, and similarly, few have any formal accountability to the public. By contrast, the architects of the pro-mask consensus are a coalition of mostly journalists, scientists, and government agencies, all of whom have established different formal systems of peer review and accountability. While it is widely known that these institutions do not always produce reliable knowledge, especially for matters concerning marginalized people (*72*–*75*), their systems for generating and vetting information are still stronger than those of a loose movement of unaffiliated conspiracy theorists and creators on alternative media platforms. As a community, scientists and publishers must work to build better, more trustworthy consensuses by strengthening both the popular perception and the actual reality of a science informed by rigorous peer review that is performed by and accountable to a diverse array of knowledgeable stakeholders (*76*), and they must understand that their work is filtered through the lens of science communicators, both friendly and unfriendly, and participate in the process of correction and communication that leads to their work being interpreted as intended.

We conclude with some comments on the construction of the datasets used in this study, which has been undoubtedly time-consuming, and how this process may be automated in the future. While automated positive/negative citation coding would allow analyses of larger controversies over longer periods of time, our experience of manually coding citations suggests that automated processes will face difficulties. We find that scientists in archival work do not criticize papers the way people criticize, for example, restaurants or movies, and will often use nongeneralizable science- and field-specific language. Criticisms can be circumspect because of admirable comity norms in science, and their meaning may be diffused over several sentences. Outside of automatic coding, simply gathering the citations in a consistent manner is likely to be quite difficult. Websites communicating important scientific information can change their content almost daily, which we observed particularly with the CDC’s websites, and this complicates attempts to understand the state of perceived scientific consensus at any given moment. Automatically collecting citations from websites with very different layouts and citation formats will also inevitably provide challenges, especially given how many of these websites do not hyperlink (or even consistently format) their citations. The popularity of video-based science communication, which combines audio content with images and text screenshots of scientific papers sometimes without titles, further increases the difficulty of completing an automatic census of communicated scientific literature as actually experienced by the media-consuming public. While we encourage research into how such data collection may be automated, we also encourage researchers to continue to engage in similar hand coding experiments in scientific communication to surface such challenges.

## MATERIALS AND METHODS

### Web of Science dataset

To understand how scientists were developing consensus around masks in the wake of the COVID-19 pandemic, we used the Web of Science paper database to collect mask-related papers in approximately the first year of the pandemic. Specifically, we collect 3593 articles from the Web of Science published between 28 March 2020 and 4 April 2021 containing in their title the following: “mask,” “personal protective equipment,” “ppe”, and “n95.” These choices were informed by familiarizing ourselves with popular papers referencing masks and the terms most frequently used to describe masks. We filter this dataset to the 82 papers that have at least 50 citations as of 18 November 2021, representing the top 2% of papers retrieved. We choose this threshold to limit the labor needed for subsequent manual coding. We exclude two papers unrelated to masking during the COVID-19 pandemic and three papers with no collectable citations, leaving a dataset of 77 papers. These papers are listed in the Supplementary Materials.

Most papers are peer-reviewed experiments and literature reviews; others are non–peer-reviewed editorials. Most directly pertain to measuring the effectiveness of masks for preventing the spread of COVID-19, but some pertain to adjacent issues such as the effectiveness of reusing masks or mitigating the waste products created from COVID-19–related mask production.

For the 77 papers, we retrieve all listed outgoing citations within the papers that are archived by the Web of Science using the digital object identifier (DOI). While this does not include all citations, our objective is to replicate those data curation practices typically performed in large-scale bibliometric studies of science. Even including citations without DOIs, the Web of Science does not include every citation that these articles make, especially when citations are made to work published outside of academic journals. There are rare instances in which the Web of Science consistently misrouted a given paper’s citation to the wrong paper, and we attempted to correct these when identified. This process results in 2426 citations, with the most cited paper as Leung *et al.* (*62*) referenced in 29 papers.

### Twitter Science Dataset

Most social media posts do not necessarily constitute scientific debate, which poses a challenge from a data collection perspective. A naive collection of Twitter posts containing the word “mask” during our study period surfaces mostly content related to sensational stories about masks. Such links rarely contain scientific content and, instead, document, for example, an altercation in a gas station started over a request to wear a mask. To discover users’ content about the effectiveness of masks, we instead specifically sample reply threads between users discussing masks, often in disagreement with one other. These arguments stimulate the trading of evidence between users in the form of external URLs, which then become the object of analysis for the Twitter Science Dataset used here.

We choose as our forum for arguments posts made in reply to the set of all Twitter accounts for U.S. governors (and the mayor of the District of Columbia) as of 28 March 2020. These political leaders are often tasked with announcing and explaining public health regulations that require wearing masks during the COVID-19 pandemic, and their posts about masks tend to generate fierce arguments between pro- and anti-mask users. From 28 March 2020 to 4 April 2021, we collect all hierarchical reply trees to these politicians that contain the word “mask” at any point in the tree. The number of posts in all reply trees totaled 37.8 million, and the subset of trees containing an instance of “mask” totaled 5.1 million posts (13% of the total). From these posts, we extracted all posts that shared a URL for a total of 158,000 unique URLs shared across 351,000 posts. We collected Twitter posts using the Twitter streaming API and independently unwound redirected links to retrieve the final destination of original URLs. Some URLs could not be retrieved because of redirect URLs no longer being operational.

From this dataset of URLs, we first remove marketing tags and other character strings appended to the end of URLs that do not affect the final landing page and then deduplicate the results. This reduces the URL dataset size by 10% to 142,000 unique URLs. To find a tractable sample for manual coding, we subsample the 194 URLs that were shared more than 100 times, which collectively are responsible for 15% of all URLs shared. Two researchers then perform a qualitative coding process on the linked content to identify which are explicitly concerned with the effectiveness (or ineffectiveness) of mask wearing for COVID-19. This restricted set of links comprises 98 unique URLs. We combined unique URLs that rehosted identical content on different websites, for a final set of 81 unique pieces of science communication. Many URLs had either been modified or, in the case of social media posts, deleted by the time of coding. To address these gaps, we use the Internet Archive (*77*) to access the most recent nonredirected version as of 4 April 2021. In only one case could a URL, linking to a removed Twitter post, not be retrieved via this method. We note that 8 of 10 of the most shared unique URLs specifically concerned themselves with a scientific analysis of the effectiveness of masks, with the remaining nonscientific links corresponding to a post by former U.S. president Donald Trump and a petition to recall California Governor Gavin Newsom.

From this dataset of 81 URLs, two researchers manually recorded every reference in the main text of these URLs. These references could be represented as embedded URLs, appended as formal academic citations, screenshots of text, or explicitly referenced by article name. In the case of URLs leading to videos, researchers watched the videos and recorded verbal citations and citations displayed in text on the video itself, a frequent behavior. All citations to external sources were recorded, including material published in scientific journals and sources such as videos, scientific journalism, and other websites. We coded the most recent version of the website as of 4 April 2021 and only content on the site that directly pertain to masks and the article in question. This resulted in 1496 citations, with the most-cited article (as in the Web of Science dataset) being that of Leung *et al.* (*62*), cited by 19 unique URLs.

### Annotating citation and website valence

In both the Web of Science and the Twitter citations datasets, the lead researcher manually annotated citations for valence, specifically noting negative citations. We define two criteria for a negative citation: (i) criticizing the methods, conclusions, or statistical power of a cited study [e.g. “Many studies in these reviews were underpowered, and most failed to measure adherence...” (*78*)] and (ii) arguing that a cited study has been popularly misinterpreted by peers or the public, either in general or for the specific case in context [e.g., “One of the most frequently mentioned, but misinterpreted, papers evaluating cloth masks...” (*79*)].

We do not label citations negatively when authors raise limitations or areas for improvement in otherwise trustworthy work or in the case where cited findings conflict with the findings of the present paper but are not themselves criticized. Our intended definition for negative citations is that, in effect, the citing article is asking readers to at least partially disregard the conclusions of a cited paper.

For our analysis of negative citation rates in Twitter science communicators, we also labeled websites in our dataset as either having a stance generally critical of masks as intervention for COVID-19 or generally positive or neutral. We considered websites to have an anti-mask orientation if they either explicitly advocated against the wearing of masks or primarily reported scientific findings supporting the ineffectiveness of masks. Anti-mask websites that had been publicly corrected and modified by the time of our analysis were considered to have neutral stance. We labeled 40 websites as critical of masks and 41 as positive or neutral.

### Network construction

Cocitation graphs are essentially undirected unipartite projections of directed bipartite graphs, where an edge between two nodes signifies that two papers are cited together in at least one citing article (*80*). Projecting a bipartite network into a unipartite network is never trivial, and choices about how to represent edges and edge weights can significantly affect resulting analyses, particularly clusterings (*81*). These choices are made even more complex in the signed case that we introduce here. We perceive three principal researcher choices with respect to these projections: (i) the choice to analyze a unipartite projection rather than the original bipartite network, (ii) the scheme for generating edges and edge weights for the unipartite projection, and (iii) the scheme for filtering edges such that communities of interest are preserved.

The first is the decision to project a bipartite graph, especially when the original graph could be considered unipartite given that some sources cite other sources in the citing set. Our choice to treat “citing” and “cited” entities as separate and, thus, bipartite stems from our understanding of this data as representing the curatorial choices of a source’s authors rather than a deep engagement with a source’s content. We find that many anti-mask communicators misleadingly cite research that actually endorses masks and itself cites extensively pro-mask literature. In directed unipartite form, this would lead to anti-mask researchers seemingly endorsing a wide variety of pro-mask literature via one transitive connection.

The second choice is how to define edges in a signed projected graph. We follow a method described in (*82*), where positive edges are considered to be (+1) and negative edges are considered to be (−1), and the weight contribution to an edge cited by two nodes being the product of their cited edges (1 × 1 = 1, −1 × −1 = 1, 1 × −1 = −1). Nodes are either together within trustworthy science (1 × 1), together within untrustworthy pseudoscience (−1 × −1), or apart from each other on either side of the boundary (1 × −1). In subsequent analysis, we analyze two such projections: one that contains all of the summed positive edge contributions (nodes cited with the same valence) and one that contains all of the negative edge contributions (nodes cited with opposite valence) over the same set of nodes. Splitting these projections, rather than summing them into one graph, aids in cluster detection via methods that optimize partitionings between multigraphs, as described in the following section.

The third choice is how to weight and filter the edges to only those relevant to the clusters at hand. As described in (*81*), projections tend to generate local structures that are highly amenable to clustering via modularity-based methods because each citing node creates a densely connected subgraph of all the nodes that it cites in the projection. Accordingly, modularity-based methods can easily return clusters that simply represent “all the papers cited by a single source.” While weighting methods have been proposed that mitigate the cluster-making ability of single sources, such as hyperbolic weight projection, we find their application to signed citation networks nontrivial and not necessarily well suited to the relatively normal degree distribution found in our data. We instead opt for a simple filtering mechanism: Connections between two papers are not considered unless they have been cited together in at least three sources in our datasets. This eliminates the cluster-making ability of single authors and single authors who create two sources and use similar citation lists. Our data does not feature authors with more than two sources, making stricter filters unnecessary. We note that this is not a simple filter on edge weights less than 3 on both subgraphs, as two sources could be cited positively together in two sources (+2) and negatively together in one (−1), and these edges would still be preserved in their respective projections because they represent cocitation in three sources.

### Clustering and visualization

Our primary clustering algorithm is based on the modularity statistic (*83*) and optimizes modularity using a multigraph method derived from the Leiden algorithm (*84*) as implemented in the Python package (*85*). Specifically, this optimization method attempts to maximize modularity on the graph representing positive ties while simultaneously minimizing modularity on the graph representing negative ties.

Historically, cocitation analyses use large, automatically collected datasets with hundreds to millions of nodes and edges. Our case is unique in that, because of hand coding, our network contains only hundreds of nodes and thousands of connections. The small size of our dataset increases the influence of noise in our data and, correspondingly, the level of uncertainty in clusterings and network representations. To address this challenge, we use a method akin to soft clustering, where individual nodes are assigned cluster probabilities rather than strict assignments, and inspired by the clustering bootstrapping procedure proposed in (*86*). Specifically, we resample each weight in the projected graphs according to a normal distribution with mean of the given weight and variance equal to the variance of all weights in the graph. We perform such replications and cluster the results 10,000 times for each graph, permuting the order of nodes provided to algorithm each time. We then create a derived representation of these graphs where each node represents a cited paper, and links between nodes are weighted by the percentage (0 to 1.0) of times these nodes were clustered in the same graph. We then apply clustering using an unsigned modularity method to this derived graph. We visualize these representations in Fig. 1, with a filter such that nodes that are clustered less than 50% of the time are removed.

We visualize graphs using the ForceAtlas2 algorithm in the network visualization software Gephi (*87*, *88*). Nodes in Fig. 1 are sized by the summed number of citations that the papers citing them have received to illustrate their importance in scientific discourse around masks in the first year of the pandemic. Nodes in Fig. 1 are similarly sized by the summed number of times the websites citing them have been shared on Twitter, for a parallel measure of importance.
