# Supplementary material for: Selective and deceptive citation in the construction of dueling consensuses
Source: Sci Adv. 2023 Sep 22;9(38):eadh1933. doi: 10.1126/sciadv.adh1933 (PMC10516490; doi:10.1126/sciadv.adh1933)
Supplement: Supplementary file 1 — Table S1 [file sciadv.adh1933_sm.pdf]

Science Advances
AAAS

Supplementary Materials for

Selective and deceptive citation in the construction of dueling consensuses

Andrew Beers *et al.*

Corresponding author: Andrew Beers, albeers@uw.edu

Sci. Adv. **9**, eadh1933 (2023)
DOI: 10.1126/sciadv.adh1933

This PDF file includes:

Table S1

Table S1

The 82 highly-cited masks papers we use in the "Web of Science" dataset are listed in the following table.

S1. S. Feng, C. Shen, N. Xia, W. Song, M. Fan, B. J. Cowling, Rational use of face masks in the COVID-19 pandemic. The Lancet Respiratory Medicine. 8, 434–436 (2020).

S2. A. Schwartz, M. Stiegel, N. Greeson, A. Vogel, W. Thomann, M. Brown, G. D. Sempowski, T. S. Alderman, J. P. Condreay, J. Burch, C. Wolfe, B. Smith, S. Lewis, Decontamination and Reuse of N95 Respirators with Hydrogen Peroxide Vapor to Address Worldwide Personal Protective Equipment Shortages During the SARS-CoV-2 (COVID-19) Pandemic. Applied Biosafety. 25, 67–70 (2020).

S3. M. L. Ranney, V. Griffeth, A. K. Jha, Critical Supply Shortages — The Need for Ventilators and Personal Protective Equipment during the Covid-19 Pandemic. New England Journal of Medicine. 382, e41 (2020).

S4. J. Xiao, E. Y. C. Shiu, H. Gao, J. Y. Wong, M. W. Fong, S. Ryu, B. J. Cowling, Nonpharmaceutical Measures for Pandemic Influenza in Nonhealthcare Settings—Personal Protective and Environmental Measures. Emerging Infectious Diseases. 26, 967–975 (2020).

S5. X. Wang, Z. Pan, Z. Cheng, Association between 2019-nCoV transmission and N95 respirator use. Journal of Hospital Infection. 105, 104–105 (2020).

S6. A. Tabah, M. Ramanan, K. B. Laupland, N. Buetti, A. Cortegiani, J. Mellinghoff, A. C. Morris, L. Camporota, N. Zappella, M. Elhadi, P. Povoa, K. Amrein, G. Vidal, L. Derde, M. Bassetti, G. Francois, N. S. yan kai, J. J. D. Waele, Personal protective equipment and intensive care unit healthcare worker safety in the COVID-19 era (PPE-SAFE): An international survey. Journal of Critical Care. 59, 70–75 (2020).

S7. E. N. Perencevich, D. J. Diekema, M. B. Edmond, Moving Personal Protective Equipment Into the Community. JAMA. 323, 2252 (2020).

S8. B. E. McGarry, D. C. Grabowski, M. L. Barnett, Severe Staffing And Personal Protective Equipment Shortages Faced By Nursing Homes During The COVID-19 Pandemic. Health Affairs. 39, 1812–1821 (2020).
S9. Y. Long, T. Hu, L. Liu, R. Chen, Q. Guo, L. Yang, Y. Cheng, J. Huang, L. Du, Effectiveness of N95 respirators versus surgical masks against influenza: A systematic review and meta-analysis. Journal of Evidence-Based Medicine. 13, 93–101 (2020).
S10. E. Livingston, A. Desai, M. Berkwits, Sourcing Personal Protective Equipment During the COVID-19 Pandemic. JAMA. 323, 1912 (2020).
S11. L. Liao, W. Xiao, M. Zhao, X. Yu, H. Wang, Q. Wang, S. Chu, Y. Cui, Can N95 Respirators Be Reused after Disinfection? How Many Times? ACS Nano. 14, 6348–6356 (2020).
S12. Z. M. Jessop, T. D. Dobbs, S. R. Ali, E. Combellack, R. Clancy, N. Ibrahim, T. H. Jovic, A. J. Kaur, A. Nijran, T. B. O'Neill, I. S. Whitaker, Personal protective equipment for surgeons during COVID-19 pandemic: systematic review of availability, usage and rationing. British Journal of Surgery. 107, 1262–1280 (2020).
S13. M. T. Hirschmann, A. Hart, J. Henckel, P. Sadoghi, R. Seil, C. Mouton, COVID-19 coronavirus: recommended personal protective equipment for the orthopaedic and trauma surgeon. Knee Surgery, Sports Traumatology, Arthroscopy. 28, 1690–1698 (2020).
S14. T. M. Cook, Personal protective equipment during the coronavirus disease (COVID) 2019 pandemic – a narrative review. Anaesthesia. 75, 920–927 (2020).
S15. J. Cohen, Y. van der M. Rodgers, Contributing factors to personal protective equipment shortages during the COVID-19 pandemic. Preventive Medicine. 141, 106263 (2020).

S16. C. Y. Benítez, A. Güemes, J. Aranda, M. Ribeiro, P. Ottolino, S. D. Saverio, H. Alexandrino, L. Ponchietti, J. L. Blas, J. P. Ramos, E. Rangelova, M. Muñoz, C. Y. and, Impact of Personal Protective Equipment on Surgical Performance During the COVID-19 Pandemic. World Journal of Surgery. 44, 2842–2847 (2020).
S17. J. J. Bartoszko, M. A. M. Farooqi, W. Alhazzani, M. Loeb, Medical masks vs N95 respirators for preventing COVID-19 in healthcare workers: A systematic review and meta-analysis of randomized trials. Influenza and Other Respiratory Viruses. 14, 365–373 (2020).
S18. Y. Wang, H. Tian, L. Zhang, M. Zhang, D. Guo, W. Wu, X. Zhang, G. L. Kan, L. Jia, D. Huo, B. Liu, X. Wang, Y. Sun, Q. Wang, P. Yang, C. R. MacIntyre, Reduction of secondary transmission of SARS-CoV-2 in households by face mask use, disinfection and social distancing: a cohort study in Beijing, China. BMJ Global Health. 5, e002794 (2020).
S19. C. Wang, A. Chudzicka-Czupa\la, D. Grabowski, R. Pan, K. Adamus, X. Wan, M. Hetna\l, Y. Tan, A. Olszewska-Guizzo, L. Xu, R. S. McIntyre, J. Quek, R. Ho, C. Ho, The Association Between Physical and Mental Health and Face Mask Use During the COVID-19 Pandemic: A Comparison of Two Countries With Different Views and Practices. Frontiers in Psychiatry. 11 (2020), doi:10.3389/fpsyt.2020.569981.
S20. R. Sommerstein, and C. A. Fux, D. Vuichard-Gysin, M. Abbas, J. Marschall, C. Balmelli, N. Troillet, S. Harbarth, M. Schlegel, A. Widmer, Risk of SARS-CoV-2 transmission by aerosols, the rational use of masks, and protection of healthcare workers from COVID-19. Antimicrobial Resistance &amp$\mathsemicolon$ Infection Control. 9 (2020), doi:10.1186/s13756-020-00763-0.

S21. J. C. Rubio-Romero, M. del C. Pardo-Ferreira, J. A. Torrecilla-García, S. Calero-Castro, Disposable masks: Disinfection and sterilization for reuse, and non-certified manufacturing, in the face of shortages during the COVID-19 pandemic. Safety Science. 129, 104830 (2020).
S22. K. A. Prather, C. C. Wang, R. T. Schooley, Reducing transmission of SARS-CoV-2. Science. 368, 1422–1424 (2020).
S23. S. Pfattheicher, L. Nockur, R. Böhm, C. Sassenrath, M. B. Petersen, The Emotional Path to Action: Empathy Promotes Physical Distancing and Wearing of Face Masks During the COVID-19 Pandemic. Psychological Science. 31, 1363–1373 (2020).
S24. R. Mittal, C. Meneveau, W. Wu, A mathematical framework for estimating risk of airborne transmission of COVID-19 with application to face mask use and social distancing. Physics of Fluids. 32, 101903 (2020).
S25. C. R. MacIntyre, A. A. Chughtai, A rapid systematic review of the efficacy of face masks and respirators against coronaviruses and other respiratory transmissible viruses for the community, healthcare workers and sick patients. International Journal of Nursing Studies. 108, 103629 (2020).
S26. W. Lyu, G. L. Wehby, Community Use Of Face Masks And COVID-19: Evidence From A Natural Experiment Of State Mandates In The US. Health Affairs. 39, 1419–1425 (2020).
S27. M. Loey, G. Manogaran, M. H. N. Taha, N. E. M. Khalifa, A hybrid deep transfer learning model with machine learning methods for face mask detection in the era of the COVID-19 pandemic. Measurement. 167, 108288 (2021).
S28. S. L. Lockhart, L. V. Duggan, R. S. Wax, S. Saad, H. P. Grocott, Personal protective equipment (PPE) for both anesthesiologists and other airway managers: principles and practice

during the COVID-19 pandemic. Canadian Journal of Anesthesia/Journal canadien d'anesthésie. 67, 1005–1015 (2020).

S29. M. Gandhi, C. Beyrer, E. Goosby, Masks Do More Than Protect Others During COVID-19: Reducing the Inoculum of SARS-CoV-2 to Protect the Wearer. Journal of General Internal Medicine. 35, 3063–3066 (2020).

S30. S. Fikenzer, T. Uhe, D. Lavall, U. Rudolph, R. Falz, M. Busse, P. Hepp, U. Laufs, Effects of surgical and FFP2/N95 face masks on cardiopulmonary exercise capacity. Clinical Research in Cardiology. 109, 1522–1530 (2020).

S31. D. K. Chu, E. A. Akl, S. Duda, K. Solo, S. Yaacoub, H. J. Schünemann, D. K. Chu, E. A. Akl, A. El-harakeh, A. Bognanni, T. Lotfi, M. Loeb, A. Hajizadeh, A. Bak, A. Izcovich, C. A. Cuello-Garcia, C. Chen, D. J. Harris, E. Borowiack, F. Chamseddine, F. Schünemann, G. P. Morgano, G. E. U. M. Schünemann, G. Chen, H. Zhao, I. Neumann, J. Chan, J. Khabsa, L. Hneiny, L. Harrison, M. Smith, N. Rizk, P. G. Rossi, P. AbiHanna, R. El-khoury, R. Stalteri, T. Baldeh, T. Piggott, Y. Zhang, Z. Saad, A. Khamis, M. Reinap, S. Duda, K. Solo, S. Yaacoub, H. J. Schünemann, Physical distancing, face masks, and eye protection to prevent person-to-person transmission of SARS-CoV-2 and COVID-19: a systematic review and meta-analysis. The Lancet. 395, 1973–1987 (2020).

S32. R. Chou, T. Dana, R. Jungbauer, C. Weeks, M. S. McDonagh, Masks for Prevention of Respiratory Virus Infections, Including SARS-CoV-2, in Health Care and Community Settings. Annals of Internal Medicine. 173, 542–555 (2020).

S33. V. C.-C. Cheng, S.-C. Wong, V. W.-M. Chuang, S. Y.-C. So, J. H.-K. Chen, S. Sridhar, K. K.-W. To, J. F.-W. Chan, I. F.-N. Hung, P.-L. Ho, K.-Y. Yuen, The role of community-

wide wearing of face mask for control of coronavirus disease 2019 (COVID-19) epidemic due to SARS-CoV-2. Journal of Infection. 81, 107–114 (2020).

S34. X. Chen, L. Ran, Q. Liu, Q. Hu, X. Du, X. Tan, Hand Hygiene, Mask-Wearing Behaviors and Its Associated Factors during the COVID-19 Epidemic: A Cross-Sectional Study among Primary School Students in Wuhan, China. International Journal of Environmental Research and Public Health. 17, 2893 (2020).

S35. J. F.-W. Chan, S. Yuan, A. J. Zhang, V. K.-M. Poon, C. C.-S. Chan, A. C.-Y. Lee, Z. Fan, C. Li, R. Liang, J. Cao, K. Tang, C. Luo, V. C.-C. Cheng, J.-P. Cai, H. Chu, K.-H. Chan, K. K.-W. To, S. Sridhar, K.-Y. Yuen, Surgical Mask Partition Reduces the Risk of Noncontact Transmission in a Golden Syrian Hamster Model for Coronavirus Disease 2019 (COVID-19). Clinical Infectious Diseases. 71, 2139–2149 (2020).

S36. C. D. Zangmeister, J. G. Radney, E. P. Vicenzi, J. L. Weaver, Filtration Efficiencies of Nanoscale Aerosol by Cloth Mask Materials Used to Slow the Spread of SARS-CoV-2. ACS Nano. 14, 9188–9200 (2020).

S37. C. J. Worby, H.-H. Chang, Face mask use in the general population and optimal resource allocation during the COVID-19 pandemic. Nature Communications. 11 (2020), doi:10.1038/s41467-020-17922-x.

S38. X. Wang, E. G. Ferro, G. Zhou, D. Hashimoto, D. L. Bhatt, Association Between Universal Masking in a Health Care System and SARS-CoV-2 Positivity Among Health Care Workers. JAMA. 324, 703 (2020).

S39. S. Ullah, A. Ullah, J. Lee, Y. Jeong, M. Hashmi, C. Zhu, K. I. Joo, H. J. Cha, I. S. Kim, Reusability Comparison of Melt-Blown vs Nanofiber Face Mask Filters for Use in the Coronavirus Pandemic. ACS Applied Nano Materials. 3, 7231–7241 (2020).

S40. A. Tcharkhtchi, N. Abbasnezhad, M. Z. Seydani, N. Zirak, S. Farzaneh, M. Shirinbayan, An overview of filtration efficiency through the masks: Mechanisms of the aerosols penetration. Bioactive Materials. 6, 106–122 (2021).
S41. N. J. Rowan, J. G. Laffey, Challenges and solutions for addressing critical shortage of supply chain for personal and protective equipment (PPE) arising from Coronavirus disease (COVID19) pandemic – Case study from the Republic of Ireland. Science of The Total Environment. 725, 138532 (2020).
S42. Q.-X. Ma, H. Shan, H.-L. Zhang, G.-M. Li, R.-M. Yang, J.-M. Chen, Potential utilities of mask-wearing and instant hand hygiene for fighting SARS-CoV-2. Journal of Medical Virology. 92, 1567–1571 (2020).
S43. M. Liang, L. Gao, C. Cheng, Q. Zhou, J. P. Uy, K. Heiner, C. Sun, Efficacy of face mask in preventing respiratory virus transmission: A systematic review and meta-analysis. Travel Medicine and Infectious Disease. 36, 101751 (2020).
S44. M. Ippolito, F. Vitale, G. Accurso, P. Iozzo, C. Gregoretti, A. Giarratano, A. Cortegiani, Medical masks and Respirators for the Protection of Healthcare Workers from SARS-CoV-2 and other viruses. Pulmonology. 26, 204–212 (2020).
S45. M. H. Haischer, R. Beilfuss, M. R. Hart, L. Opielinski, D. Wrucke, G. Zirgaitis, T. D. Uhrich, S. K. Hunter, Who is wearing a mask? Gender-, age-, and location-related differences during the COVID-19 pandemic. PLOS ONE. 15, e0240785 (2020).
S46. B. Ghanbari, A. Atangana, A new application of fractional Atangana–Baleanu derivatives: Designing ABC-fractional masks in image processing. Physica A: Statistical Mechanics and its Applications. 542, 123516 (2020).

S47. S. Esposito, N. Principi, C. C. Leung, G. B. Migliori, Universal use of face masks for success against COVID-19: evidence and implications for prevention policies. European Respiratory Journal. 55, 2001260 (2020).
S48. N. El-Atab, N. Qaiser, H. Badghaish, S. F. Shaikh, M. M. Hussain, Flexible Nanoporous Template for the Design and Development of Reusable Anti-COVID-19 Hydrophobic Face Masks. ACS Nano. 14, 7659–7665 (2020).
S49. S. Bae, M.-C. Kim, J. Y. Kim, H.-H. Cha, J. S. Lim, J. Jung, M.-J. Kim, D. K. Oh, M.-K. Lee, S.-H. Choi, M. Sung, S.-B. Hong, J.-W. Chung, S.-H. Kim, Effectiveness of Surgical and Cotton Masks in Blocking SARS–CoV-2: A Controlled Comparison in 4 Patients. Annals of Internal Medicine. 173, W22–W23 (2020).
S50. S. Asadi, C. D. Cappa, S. Barreda, A. S. Wexler, N. M. Bouvier, W. D. Ristenpart, Efficacy of masks and face coverings in controlling outward aerosol particle emission from expiratory activities. Scientific Reports. 10 (2020), doi:10.1038/s41598-020-72798-7.
S51. T. A. Aragaw, Surgical face masks as a potential source for microplastic pollution in the COVID-19 scenario. Marine Pollution Bulletin. 159, 111517 (2020).
S52. P. C. Addo, F. Jiaming, N. B. Kulbo, L. Liangqiang, COVID-19: fear appeal favoring purchase behavior towards personal protective equipment. The Service Industries Journal. 40, 471–490 (2020).
S53. H. Zhong, Z. Zhu, J. Lin, C. F. Cheung, V. L. Lu, F. Yan, C.-Y. Chan, G. Li, Reusable and Recyclable Graphene Masks with Outstanding Superhydrophobic and Photothermal Performances. ACS Nano. 14, 6213–6221 (2020).
S54. S. Verma, M. Dhanak, J. Frankenfield, Visualizing droplet dispersal for face shields and masks with exhalation valves. Physics of Fluids. 32, 091701 (2020).

S55. S. Verma, M. Dhanak, J. Frankenfield, Visualizing the effectiveness of face masks in obstructing respiratory jets. Physics of Fluids. 32, 061708 (2020).

S56. H. Ueki, Y. Furusawa, K. Iwatsuki-Horimoto, M. Imai, H. Kabata, H. Nishimura, Y. Kawaoka, Effectiveness of Face Masks in Preventing Airborne Transmission of SARS-CoV-2. mSphere. 5 (2020), doi:10.1128/msphere.00637-20.

S57. A. Pezzini, A. Padovani, Lifting the mask on neurological manifestations of COVID-19. Nature Reviews Neurology. 16, 636–644 (2020).

S58. L. J. Mady, M. W. Kubik, K. Baddour, C. H. Snyderman, N. R. Rowan, Consideration of povidone-iodine as a public health intervention for COVID-19: Utilization as "Personal Protective Equipment" for frontline providers exposed in high-risk head and neck and skull base oncology care. Oral Oncology. 105, 104724 (2020).

S59. X. Liu, S. Zhang, COVID-19: Face masks and human-to-human transmission. Influenza and Other Respiratory Viruses. 14, 472–473 (2020).

S60. N. H. L. Leung, D. K. W. Chu, E. Y. C. Shiu, K.-H. Chan, J. J. McDevitt, B. J. P. Hau, H.-L. Yen, Y. Li, D. K. M. Ip, J. S. M. Peiris, W.-H. Seto, G. M. Leung, D. K. Milton, B. J. Cowling, Respiratory virus shedding in exhaled breath and efficacy of face masks. Nature Medicine. 26, 676–680 (2020).

S61. C. C. Leung, T. H. Lam, K. K. Cheng, Mass masking in the COVID-19 epidemic: people need guidance. The Lancet. 395, 945 (2020).

S62. A. Konda, A. Prakash, G. A. Moss, M. Schmoldt, G. D. Grant, S. Guha, Aerosol Filtration Efficiency of Common Fabrics Used in Respiratory Cloth Masks. ACS Nano. 14, 6339–6347 (2020).

S63. J. Howard, A. Huang, Z. Li, Z. Tufekci, V. Zdimal, H.-M. van der Westhuizen, A. von Delft, A. Price, L. Fridman, L.-H. Tang, V. Tang, G. L. Watson, C. E. Bax, R. Shaikh, F. Questier, D. Hernandez, L. F. Chu, C. M. Ramirez, A. W. Rimoin, An evidence review of face masks against COVID-19. Proceedings of the National Academy of Sciences. 118 (2021), doi:10.1073/pnas.2014564118.
S64. T. Greenhalgh, M. B. Schmid, T. Czypionka, D. Bassler, L. Gruer, Face masks for the public during the covid-19 crisis. BMJ, m1435 (2020).
S65. M. Gandhi, G. W. Rutherford, Facial Masking for Covid-19 — Potential for “Variolation” as We Await a Vaccine. New England Journal of Medicine. 383, e101 (2020).
S66. E. P. Fischer, M. C. Fischer, D. Grass, I. Henrion, W. S. Warren, E. Westman, Low-cost measurement of face mask efficacy for filtering expelled droplets during speech. Science Advances. 6 (2020), doi:10.1126/sciadv.abd3083.
S67. O. O. Fadare, E. D. Okoffo, Covid-19 face masks: A potential source of microplastic fibers in the environment. Science of The Total Environment. 737, 140279 (2020).
S68. M. H. Chua, W. Cheng, S. S. Goh, J. Kong, B. Li, J. Y. C. Lim, L. Mao, S. Wang, K. Xue, L. Yang, E. Ye, K. Zhang, W. C. D. Cheong, B. H. Tan, Z. Li, B. H. Tan, X. J. Loh, Face Masks in the New COVID-19 Normal: Materials, Testing, and Perspectives. Research. 2020, 1–40 (2020).
S69. H. Bundgaard, J. S. Bundgaard, D. E. T. Raaschou-Pedersen, C. von Buchwald, T. Todsen, J. B. Norsk, M. M. Pries-Heje, C. R. Vissing, P. B. Nielsen, U. C. Winsløw, K. Fogh, R. Hasselbalch, J. H. Kristensen, A. Ringgaard, M. P. Andersen, N. B. Goecke, R. Trebbien, K. Skovgaard, T. Benfield, H. Ullum, C. Torp-Pedersen, K. Iversen, Effectiveness of Adding a

Mask Recommendation to Other Public Health Measures to Prevent SARS-CoV-2 Infection in Danish Mask Wearers. Annals of Internal Medicine. 174, 335–343 (2021).

S70. J. T. Brooks, J. C. Butler, R. R. Redfield, Universal Masking to Prevent SARS-CoV-2 Transmission—The Time Is Now. JAMA. 324, 635 (2020).

S71. G. R. J. Swennen, L. Pottel, P. E. Haers, Custom-made 3D-printed face masks in case of pandemic crisis situations with a lack of commercially available FFP2/3 masks. International Journal of Oral and Maxillofacial Surgery. 49, 673–677 (2020).

S72. A. L. P. Silva, J. C. Prata, T. R. Walker, D. Campos, A. C. Duarte, A. M. V. M. Soares, D. Barcelò, T. Rocha-Santos, Rethinking and optimising plastic waste management under COVID-19 pandemic: Policy solutions based on redesign and reduction of single-use plastics and personal protective equipment. Science of The Total Environment. 742, 140565 (2020).

S73. S. W. X. Ong, Y. K. Tan, P. Y. Chia, T. H. Lee, O. T. Ng, M. S. Y. Wong, K. Marimuthu, Air, Surface Environmental, and Personal Protective Equipment Contamination by Severe Acute Respiratory Syndrome Coronavirus 2 (SARS-CoV-2) From a Symptomatic Patient. JAMA. 323, 1610 (2020).

S74. P. Mick, R. Murphy, Aerosol-generating otolaryngology procedures and the need for enhanced PPE during the COVID-19 pandemic: a literature review. Journal of Otolaryngology - Head &amp$\mathsemicolon$ Neck Surgery. 49 (2020), doi:10.1186/s40463-020-00424-7.

S75. T. Li, Y. Liu, M. Li, X. Qian, S. Y. Dai, Mask or no mask for COVID-19: A public health and market study. PLOS ONE. 15, e0237691 (2020).

S76. M. Klompas, C. A. Morris, J. Sinclair, M. Pearson, E. S. Shenoy, Universal Masking in Hospitals in the Covid-19 Era. New England Journal of Medicine. 382, e63 (2020).

S77. J. J. Klemeš, Y. V. Fan, P. Jiang, The energy and environmental footprints of COVID-19 fighting measures – PPE, disinfection, supply chains. Energy. 211, 118701 (2020).
S78. B. Javid, M. P. Weekes, N. J. Matheson, Covid-19: should the public wear face masks? BMJ, m1442 (2020).
S79. T. Dbouk, D. Drikakis, On respiratory droplets and face masks. Physics of Fluids. 32, 063303 (2020).
S80. J. J. Y. Ong, C. Bharatendu, Y. Goh, J. Z. Y. Tang, K. W. X. Sooi, Y. L. Tan, B. Y. Q. Tan, H.-L. Teoh, S. T. Ong, D. M. Allen, V. K. Sharma, Headaches Associated With Personal Protective Equipment – A Cross-Sectional Study Among Frontline Healthcare Workers During COVID-19. Headache: The Journal of Head and Face Pain. 60, 864–877 (2020).
S81. M. Liu, S.-Z. Cheng, K.-W. Xu, Y. Yang, Q.-T. Zhu, H. Zhang, D.-Y. Yang, S.-Y. Cheng, H. Xiao, J.-W. Wang, H.-R. Yao, Y.-T. Cong, Y.-Q. Zhou, S. Peng, M. Kuang, F.-F. Hou, K. K. Cheng, H.-P. Xiao, Use of personal protective equipment against coronavirus disease 2019 by healthcare professionals in Wuhan, China: cross sectional study. BMJ, m2195 (2020).
S82. R. J. Fischer, D. H. Morris, N. van Doremalen, S. Sarchette, M. J. Matson, T. Bushmaker, C. K. Yinda, S. N. Seifert, A. Gamble, B. N. Williamson, S. D. Judson, E. de Wit, J. O. Lloyd-Smith, V. J. Munster, Effectiveness of N95 Respirator Decontamination and Reuse against SARS-CoV-2 Virus. Emerging Infectious Diseases. 26, 2253–2255 (2020).
